# Evaluation and optimisation of indel detection workflows for ion torrent sequencing of the *BRCA1* and *BRCA2* genes

**DOI:** 10.1186/1471-2164-15-516

**Published:** 2014-06-24

**Authors:** Zhen Xuan Yeo, Joshua Chee Leong Wong, Steven G Rozen, Ann Siew Gek Lee

**Affiliations:** Division of Medical Sciences, National Cancer Centre Singapore, Singapore, Singapore; Centre for Computational Biology, Duke-NUS Graduate Medical School, Singapore, Singapore; Office of Clinical & Academic Faculty Affairs, Duke-NUS Graduate Medical School, Singapore, Singapore; Department of Physiology, Yong Loo Lin School of Medicine, National University of Singapore, Singapore, Singapore

**Keywords:** Mutation detection, Indels, Next generation sequencing, *BRCA1*, *BRCA2*, Ion Torrent, Variant calling, Workflow

## Abstract

**Background:**

The Ion Torrent PGM is a popular benchtop sequencer that shows promise in replacing conventional Sanger sequencing as the gold standard for mutation detection. Despite the PGM’s reported high accuracy in calling single nucleotide variations, it tends to generate many false positive calls in detecting insertions and deletions (indels), which may hinder its utility for clinical genetic testing.

**Results:**

Recently, the proprietary analytical workflow for the Ion Torrent sequencer, Torrent Suite (TS), underwent a series of upgrades. We evaluated three major upgrades of TS by calling indels in the *BRCA1* and *BRCA2* genes. Our analysis revealed that false negative indels could be generated by TS under both default calling parameters and parameters adjusted for maximum sensitivity. However, indel calling with the same data using the open source variant callers, GATK and SAMtools showed that false negatives could be minimised with the use of appropriate bioinformatics analysis. Furthermore, we identified two variant calling measures, Quality-by-Depth (QD) and VARiation of the Width of gaps and inserts (VARW), which substantially reduced false positive indels, including non-homopolymer associated errors without compromising sensitivity. In our best case scenario that involved the TMAP aligner and SAMtools, we achieved 100% sensitivity, 99.99% specificity and 29% False Discovery Rate (FDR) in indel calling from all 23 samples, which is a good performance for mutation screening using PGM.

**Conclusions:**

New versions of TS, BWA and GATK have shown improvements in indel calling sensitivity and specificity over their older counterpart. However, the variant caller of TS exhibits a lower sensitivity than GATK and SAMtools. Our findings demonstrate that although indel calling from PGM sequences may appear to be noisy at first glance, proper computational indel calling analysis is able to maximize both the sensitivity and specificity at the single base level, paving the way for the usage of this technology for future clinical genetic testing.

**Electronic supplementary material:**

The online version of this article (doi:10.1186/1471-2164-15-516) contains supplementary material, which is available to authorized users.

## Background

Dideoxynucleotide sequencing of DNA or Sanger sequencing has been the gold standard for mutation screening for over two decades. In recent years, the emergence of benchtop next generation sequencing (NGS) has offered a powerful alternative for mutation detection. Compared to Sanger sequencing, benchtop NGS can detect mutations from a significantly larger number of samples in parallel, in a more cost effective manner [1, 2]. Nevertheless, several studies have compared currently available benchtop sequencers to determine their mutation detection accuracy [3–5]. These studies have highlighted that the accuracy of mutation detection may need to be improved in order for NGS to become a prudent option for clinical genetic testing [1, 6].

**Figure 1:**
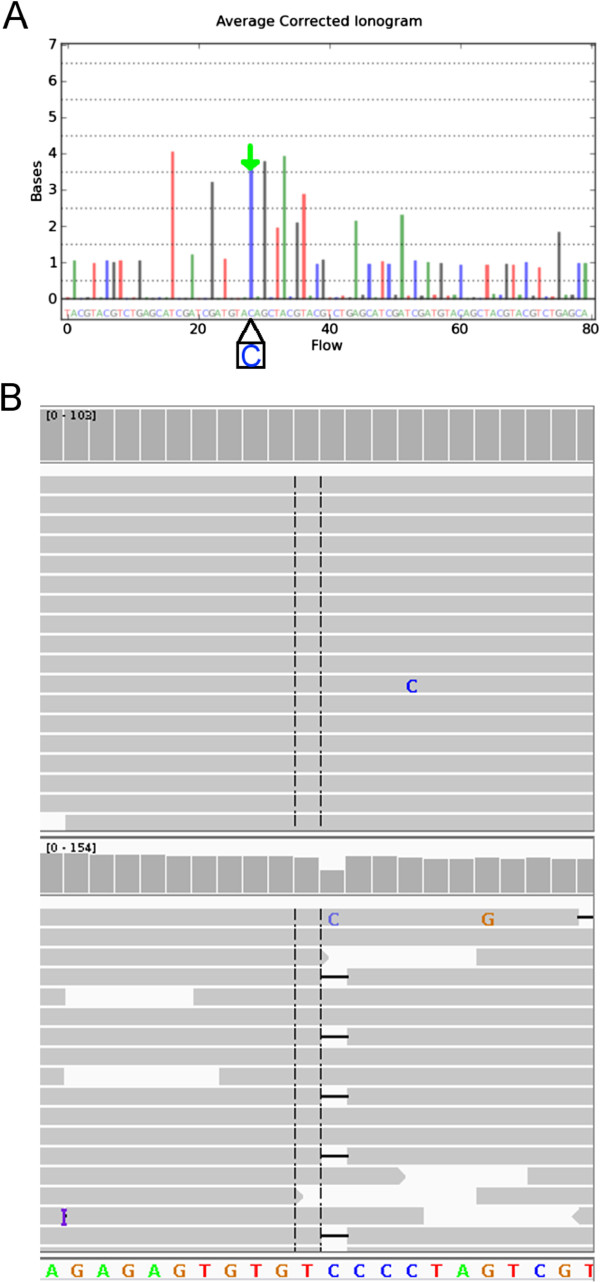
**Examples to illustrate base calling errors associated with homopolymers generated by PGM sequencing. A**: An example of a homopolymer indel error illustrated with the PGM Ionogram. An ionogram is a graphical representation that demonstrates the conversion of PGM sequencing output to read sequences. The x-axis indicates the nucleotides along the read sequence. The y-axis indicates the number of consecutively identical nucleotide. One peak in the ionogram (arrowed) has a peak height of between three and four 'C' bases which suggests that the read sequence at this region could be ‘CCC’ or ‘CCCC’. During read alignment, if the reference sequence has four 'C' bases in this region, a deletion might be generated by reads with three 'C' bases. **B**: The top panel represents an IGV snapshot that indicates the read alignment of a DNA region with no indel generated by SOLiD sequencing. The bottom panel shows a “deletion” detected using PGM resequencing for the same region as the top panel.

The Ion Torrent PGM is a semiconductor based benchtop DNA sequencer, which was launched in 2011. The PGM generates DNA sequencing reads by detecting ions released when deoxribonucleotide triphospates are incorporated into a growing DNA strand on a semiconductor device [7]. A growing number of studies have utilized the PGM to detect genetic variation associated with human diseases [2, 8–10]. In general, the PGM performed well in accurately detecting single nucleotide variations (SNV) but the overall specificity remained low due to the high false positive rate for indel detection [2, 5, 6]. In particular, it has been documented that indel errors occurring in homopolymer DNA regions have significantly affected the specificity of indel detection [3, 6, 11, 12]. Due to the nature of the sequencing chemistry of PGM, any genomic region with consecutively identical DNA bases (a homopolymer region) will have a higher indel calling error rate than other genomic regions, as a result of uncertainty in determining the signal intensity that represents the precise number of homopolymer bases (Figure 1). For clinical genetic tests, a low false positive rate is necessary if the clinical laboratory is screening for deleterious mutations using the PGM for tens or hundreds of patients.

To rectify the problem of high false positive rates for indel detection, since late 2012 Ion Torrent has conducted multiple upgrades of the Torrent Suite (TS), the proprietary analytical workflow for the Ion Torrent benchtop sequencers. A more recent version of the Torrent Suite Variant Caller (TSVC) uses BAM files which include flow signal information (‘FZ’ tag in BAM file as defined by SAM format [13]). In theory, the use of flow signal information is expected to improve the accuracy of variant calling. It is currently unknown if these upgrades affect the specificity and sensitivity of variant detection in clinical data and how the TSVC performs when compared to open source variant callers that do not consider flow signal information.

Recently, Costa *et al.* presented a workflow for use of the Ion Torrent PGM for clinical genetic testing of the *BRCA1* and *BRCA2* genes [2], achieving ≥ 98.6% sensitivity and only 96.9% specificity, which is low for high-throughput clinical diagnostics. The workflow used a filter of 'variants < 15% cases' which requires prior understanding of genetic diversity of the given samples and which may result in low sensitivity for samples derived from family members or that contain highly conserved functional mutations. Therefore, a better strategy that offers a similar or better sensitivity and specificity without the need of such a filtering criterion is warranted.

Indel detection accuracy is highly dependent on the bioinformatics data processing pipeline and the selection of parameters within the variant calling software [6]. Our previous study on improving the indel detection specificity of *BRCA1* and *BRCA2* genes using PGM implemented two simple filtering criteria: B-allele frequency (BAF) and VARiation of the Width of gaps and inserts (VARW) [6]. These allowed us to achieve ≥ 99.99% specificity and yet retaining 100% sensitivity. However, the estimation of BAF is unreliable in regions of low read depth [14]. An alternative to the BAF threshold – an alternative that is independent of read depth would be useful to further improve detection specificity.

The aims of this present study are first to compare the performance of the PGM platform using TS versions 2.0, 2.2 and 3.4 for indel detection of the *BRCA1* and *BRCA2* genes. Second, we evaluated one open source read mapper (BWA) and two open source variant callers (GATK and SAMtools) to find out if they are suitable or better in calling indels. In addition, we report here that two measures, Quality by Depth (QD) and VARW, can substantially improve detection specificity without compromising sensitivity. A bioinformatics workflow for indel detection from our *BRCA1* and *BRCA2* dataset is proposed; this workflow does not require any prior knowledge of the genetic diversity of given samples. After developing the workflow, we validated it in an independent set of 17 samples.

## Methods

### Sample preparation and DNA sequencing

DNA sample preparation, Sanger and Ion Torrent PGM sequencing were performed as described previously [1]. Briefly, blood samples were obtained from patients attending outpatient clinics at the National Cancer Centre Singapore with written informed consent. Ethics approval for this study was obtained from the Centralized Institutional Review Board of SingHealth (Singapore). DNA was extracted using an optimized in-house method [15].

The complete coding regions of *BRCA1* and *BRCA2*, with ~40 bp of non-coding sequence flanking the 5' and 3' ends of each exon, were PCR amplified [1]. The amplicons, spanning 25,427 bp of non-overlapping regions were Sanger sequenced, with sequence alignment and variant detection carried out using SeqMan Pro from Lasergene 8.0 (DNASTAR, Inc., US).

The same PCR amplicons were used for sequencing on the Ion Torrent PGM. Fragment libraries were constructed by DNA fragmentation, barcode and adaptor ligation, library amplification, and fragment size selection using agarose gel electrophoresis. Template preparation, emulsion PCR, and Ion Sphere Particles (ISP) enrichment were carried out using the Ion Xpress Template kit (Life Technologies Corp., US). The quality of the ISPs was assessed using a Qubit 2.0 Fluorometer (Life Technologies), and the ISPs were loaded and sequenced on a 316 chip (Life Technologies). The PGM sequencing run outputs were directly loaded to the Torrent Server and stored as ‘.dat’ files.

### Read mapping

We evaluated 4 read mappers (Table 1): mappers of Torrent Suite (TS) 2.0, TS2.2 and TS3.4 (denoted as TMAP-TS2.0, 2,2 and 3.4), as well as Burrows-Wheeler Aligner (BWA, version 0.6.2, [16]). We downloaded Torrent Virtual Machines containing the different versions of TS (TS2.0, 2.2 and 3.4) from the Ion Community. We downloaded BWA from http://bio-bwa.sourceforge.net/. The FASTQ files required as input by BWA were generated automatically by TS2.0. Read mappers were run with default parameters unless stated otherwise. For BWA, ‘bwasw’ was set as the algorithm for the read mapping. hg19 was used as the reference genome.

**Table 1:** **Comparison of indel calling in the 6 training samples using different variant calling workflows, without subsequent filtering**

Read mapper	Variant caller	FP ^a^	FN ^a^	TP ^a^	TN ^a^	Sensitivity [95% CI]	Specificity [95% CI]	FDR [95% CI]
TMAP-TS2.0	TSVC2.0	0	2	1	96135	33.33% [3.87, 82.33]	100% [100, 100]	0% [0, 77.15]
TMAP-TS2.2	TSVC2.2	0	2	1	96135	33.33% [3.87, 82.33]	100% [100, 100]	0% [0, 77.15]
TMAP-TS3.4	TSVC3.4	8	1	2	96127	66.67% [17.67, 96.13]	99.99% [99.98, 100]	80% [49.72, 95.59]
TMAP-TS2.0	GATK	4	1	2	96131	66.67% [17.67, 96.13]	99.99% [99.99, 100]	66.67% [28.64, 92.32]
TMAP-TS2.2	GATK	9	1	2	96126	66.67% [17.67, 96.13]	99.99% [99.98, 100]	81.82% [53.28, 96.02]
*TMAP-TS3.4	GATK	5	0	3	96130	100% [55.59, 100]	99.99% [99.99, 100]	62.5% [29.48, 88.1]
TMAP-TS2.0	SAMtools	0	3	0	96135	100% [55.59, 100]	99.97% [99.96, 99.98]	90.62% [77.05, 97.29]
TMAP-TS2.2	SAMtools	39	3	0	96096	100% [55.59, 100]	99.99% [99.98, 99.99]	81.25% [57.92, 94.42]
*TMAP-TS3.4	SAMtools	17	0	3	96118	100% [55.59, 100]	99.98% [99.97, 99.99]	85% [65.14, 95.59]
*BWA	GATK	1	0	3	96134	100% [55.59, 100]	99.99% [99.99, 100]	25% [2.85, 71.62]
*BWA	SAMtools	20	0	3	96115	100% [55.59, 100]	99.98% [99.97, 99.99]	86.96% [69.13, 96.19]

We considered all bases in coding exons. Across the 6 samples the total number of bases considered was 96,138.

^a^FP = False Positives; FN = False Negatives; TP = True Positive; TN = True Negatives.

*Workflow with 100% sensitivity.

### Indel calling

We evaluated five indel callers in various combinations with read mappers, as shown in Table 1. Briefly, we used the indel callers from TS2.0, TS2.2, and TS3.4 (indel callers denoted TSVC2.0, TSVC2.2, and TSVC3.4, respectively), as well as the indel callers from GATK version 2.3-6 (UnifiedGenotyper) [17] and SAMtools 1.1.18 (mpileup and bcftools) [13]. Default parameters were used when TS2.0, TS2.2 and TS3.4 were applied for indel calling. For indel calling using GATK2.3-6 and SAMtools1.1.18, the raw BAM files were preprocessed according to GATK's NGS data preprocessing workflow [17] where deduplication, local realignment and base quality recalibration were performed. For indels called by GATK2.3-6, GATK's VariantFiltration was applied to remove potential false positives indicated by strand bias, errors at the ends of reads and low read depth. When applying GATK and SAMtools, selected parameters were modified to achieve high sensitivity. For GATK, we set stand_call_conf = 10 and stand_emit_conf = 10. For SAMtools, we set homopolymer coefficient h = 50. The RefSeq coding exons of *BRCA1* and *BRCA2* genes were defined as ‘callable’ regions which covered 16023 bp of non-overlapping region.

## Results

### Performance evaluation of the torrent suite for indel detection

The performance of mutation detection in *BRCA1* and *BRCA2* using multiple combinations of read mappers and variant callers was evaluated in six germ-line DNA samples (Table 1). We compared the PGM results to results from "gold standard" Sanger sequencing of the same PCR products. Three 'true' indels (*BRCA2:NM_000059:c.3846_3847del*, *BRCA2:NM_000059:c.7696_7697insA*, *BRCA1:NM_007294c.3424delG*) specific to three different samples were identified by Sanger sequencing. Variant calling using combination of TMAP-TS and TSVC generated a range of sensitivity of between 33.3%-66.6%, a range of specificity of between 99%-100% and a range of FDR of between 0%-90.6% (Table 1). Table 1 shows an improvement in sensitivity for version 3.4 as compared to the older versions, 2.0/2.2 (when using their TAMP-TSs and TSVCs). All three versions missed one indel (*BRCA2:NM_000059:c.7696_7697insA*), and 2.0/2.2 missed an additional indel (*BRCA2:NM_000059:c.3846_3847del*).

To summarize the finding for the three TS versions, the TMAP-TS3.4 + TSVC3.4 combination had substantially better sensitivity than the other two, but with a decrease in specificity (99.99% as opposed to 100% for 2.0 and 2.2, Table 1) and a higher FDR (80% as opposed to 0% for 2.0 and 2.2, Table 1).

### Impact of mapping quality on detection sensitivity

It is possible that mapping quality – the accuracy with which reads are mapped to the correct location in the reference genome – could affect detection sensitivity. We examined two false negatives in the light of this possibility.

The failure of TMAP-TS + TSVC2.0/2.2 to detect one true positive (*BRCA2:NM_000059:c.3846_3847del*) might have been a consequence of inaccurate mapping. To explore this possibility, we examined the MAPQ (“MAPping Quality) scores in TS2.0/2.2 versus TS3.4 alignments. MAPQ indicates whether a read is likely to be mapped to the correct location [18], with high values indicating good read mapping. The TMAP-TS + TSVC3.4 generated a MAPQ distribution with higher median values (median MAPQ = 66) than that of TMAP-TS + TSVC2.0 (median MAPQ = 26) and TMAP-TS + TSVC 2.2 (median MAPQ = 47) (Additional file 1: Figure S3). We also manually inspected the alignment (Additional file 1: Figure S1) using IGV [19, 20]. In comparison to the TS3.4 alignments, TS2.0/2.2 alignments contain more mismatches, exhibit higher variation in size and have more erroneous gaps proximal to the indel position (Additional file 1: Figure S1). These observations combined with the MAPQ distributions suggest that this false negative is possibly associated with reads mapped to incorrect locations.

As highlighted in the previous section, one true positive indel (*BRCA2:NM_000059:c.7696_7697insA*) was missed by all TS versions. However, it was noticed that similar median MAPQ values and MAPQ distributions were generated by TS2.2 and TS3.4 at this position (Additional file 1: Figure S4). By manual inspection using IGV, we observed relatively high coverage (>40X), sufficient non-reference allele frequency (>0.28) and clean alignment profile (Additional file 1: Figure S2) at this indel position. The IGV inspection, taken together with the MAPQ scores suggest that this false negative indel call was not due to read mapping errors.

### Variant calling from PGM data using GATK and SAMtools

The three TS variant callers were unable to achieve 100% sensitivity, as shown in the previous analysis. To investigate whether applying alternative variant callers would improve the sensitivity, we also assessed two alternative, widely-used variant callers, GATK and SAMtools, on the *BRCA1* and *BRCA2* data.

Both GATK and SAMtools achieved 100% sensitivity and 99% specificity on alignment data generated by TS3.4 (Table 1). GATK also performed better than TSVC when calling indels from alignment data of TS2.2. Along with higher sensitivity, both GATK and SAMtools had a lower specificity than TSVC.

To determine if the indel not detected by TSVC (*BRCA2:NM_000059:c.7696_7697insA*) was due to the trade-off between sensitivity and specificity, we re-ran the variant calling with adjusted TS3.4 variant calling parameters, in which the calling sensitivity was maximized (Additional file 1: Table S1). The single missed true positive indel (*BRCA2:NM_000059:c.7696_7697insA*) remained undetected, which suggested that GATK and SAMtools were more sensitive than TSVC.

GATK and SAMtools were also used to call indels from alignment data generated by the, BWA mapper. Median MAPQs at all three true positive indel positions were lower in BWA-generated alignments (Additional file 1: Figure S3 and Figure S4). Nonetheless, the sensitivity of indel calling using both GATK and SAMtools remained as 100% (Table 1).

### Characteristics of false positive variants detected by TSVC, GATK and SAMtools

The previous analyses highlighted that variant calling using TSVC2.0, 2.2 and 3.4 showed a problem with sensitivity (Table 1). Without any clear avenues to improve their sensitivities, we focused on improving the specificity of indel calling by using GATK and SAMtools, the variant callers in our study that had 100% sensitivity using alignments from either TMAP-TS3.4 or BWA.

Although GATK and SAMtools were able to call variants with 100% sensitivity when applied to BAM files generated by TMAP-TS3.4 and BWA, false positive indels remained detected in the six samples, with some of these workflows generating up to 20 false positives (Table 1). To explore the utility of potential approaches to reducing the number of false positives, in the context of TSVC3.4, GATK and SAMtools, we compared the distributions of four measurements associated with false positive and true positive indels. These measurements were B-allele frequency (BAF), Quality score of called variant (QUAL), Quality by depth (QD) and VARiation of the Width of gaps and inserts (VARW).

B-allele frequency (BAF) represents the proportion of reads with the non-reference allele. The QUALity scores of called variants (QUAL) were generated by the variant callers and were provided in their output VCF files. Quality by depth (QD) was computed through the division of QUAL by read depth. VARiation of the Width of gaps and inserts (VARW) was calculated as described in our previous work [6].

**Figure 2:**
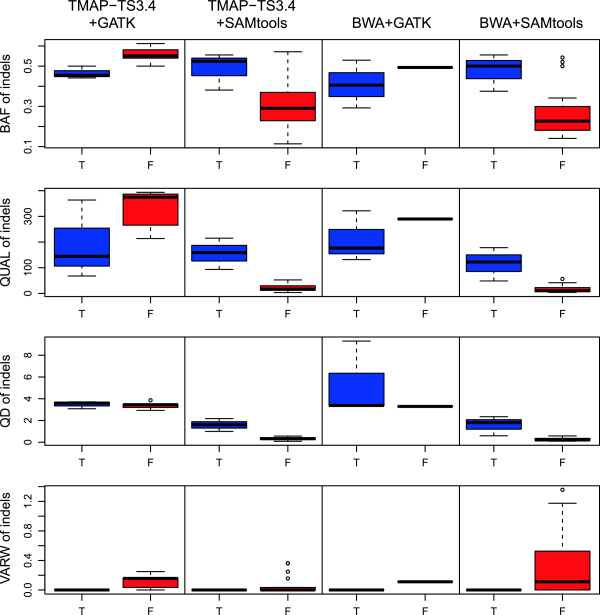
**Characteristics of true (T) and false (F) positive indels.** Four panels show the boxplot distributions of BAF, QUAL, QD and VARW for true (blue) and false (red) positive indels detected by different indel calling workflows indicated at the top of the panels. The false positive indels detected by workflows using GATK as variant caller show higher average BAF and average QUAL than the values of true positive indels. Only QD and VARW had a consistent trend detected by all workflows, with true positive indels having a higher average QD and lower average VARW than the values of false positive indels.

We examined the distribution of the four measurements generated by indel calling workflows that used the TMAP-TS3.4 and BWA alignments combined with the GATK and SAMtools variant calling. The selected workflows were denoted as ‘TMAP-TS3.4 + GATK’, ‘TMAP-TS3.4 + SAMtools’, ‘BWA + GATK’ and ‘BWA + SAMtools’ (Figure 2).

### Improvement of variant calling specificity using VARW threshold and QD threshold

Analyzing the characteristics of true and false positive indels (Figure 2) suggested that QD and VARW could be used to distinguish the true and false positive indels. QD thresholds (QD_th_) and VARW thresholds (VARW_th_) specific to GATK (QD_th_ = 2.5, VARW_th_ = 0) and SAMtools (QD_th_ = 1, VARW_th_ = 0) were applied to indels called by the four workflows with 100% sensitivity (Table 1). The threshold values were selected to achieve maximum sensitivity and specificity based on the analysis of the distributions of QD and VARW (Figure 2). QD_th_ differed between the GATK and SAMtools due to the different QUAL scores generated by the two variant callers.

Using these QD_th_ and VARW_th_ filters improved the specificity of indel calling from the workflows, with ≤ 1 false positive indel detected by the four workflows (Table 2). Indeed, three of four workflows achieved 100% sensitivity and specificity, and 0% FDR when QD_th_ and VARW_th_ filters were used.

**Table 2:** **Comparison of indel calling in the 6 training samples using different workflows with QD**_**th**_ **and VARW**_**th**_ **filters**

Read mapper	Variant caller	QD _th_	VARW _th_	FP ^a^	FN ^a^	TP ^a^	TN ^a^	Sensitivity [95% CI]	Specificity [95% CI]	FDR [95% CI]
TMAP-TS3.4	GATK	2.5	0	1	0	3	96134	100% [55.59, 100]	99.99% [99.99, 100]	25% [2.85, 71.62]
TMAP-TS3.4	SAMtools	1	0	0	0	3	96135	100% [55.59, 100]	100% [100, 100]	0% [0, 44.41]
BWA	GATK	2.5	0	0	0	3	96135	100% [55.59, 100]	100% [100, 100]	0% [0, 44.41]
BWA	SAMtools	1	0	0	0	3	96135	100% [55.59, 100]	100% [100, 100]	0% [0, 44.41]

We considered all bases in coding exons. Across the 6 samples the total number of bases considered was 96,138.

^a^FP = False Positives; FN = False Negatives; TP = True Positive; TN = True Negatives.

### Validation of the workflows and filters

We evaluated the four workflows (Table 2) on a test set of 17 additional samples with unknown mutation status. In addition to PGM sequencing, Sanger sequencing of the 17 samples was performed to determine their true mutation status.

The four workflows differed in the number of false positives, but all achieved ≥ 99.99% specificity (Table 3). When using the alignments generated by TMAP-TS3.4 and BWA for indel calling, the SAMtools variant caller performed best, with 2 (FDR = 50%) and 4 (FDR = 66.7%) false positives respectively (Table 3). GATK detected 25 (FDR = 92.6%) and 14 (FDR = 87.5%) false positives respectively.

**Table 3:** **Comparison of indel calling in the 17 additional test samples using different workflows with QD**_**th**_ **and VARW**_**th**_ **filters**

Read mapper	Variant caller	QD _th_	VARW _th_	FP ^a^	FN ^a^	TP ^a^	TN ^a^	Sensitivity [95% CI]	Specificity [95% CI]	FDR [95% CI]
TMAP-TS3.4	GATK	2.5	0	25	0	2	272364	100% [43.07, 100]	99.99% [99.99, 99.99]	92.59% [78.3, 98.43]
TMAP-TS3.4	SAMtools	1	0	2	0	2	272387	100% [43.07, 100]	99.99% [99.99, 100]	50% [12.28, 87.72]
BWA	GATK	2.5	0	14	0	2	272375	100% [43.07, 100]	99.99% [99.99, 100]	87.5% [65.58, 97.31]
BWA	SAMtools	1	0	4	0	2	272385	100% [43.07, 100]	99.99% [99.99, 100]	66.67% [28.64, 92.32]

We considered all bases in coding exons. Across the 17 samples the total number of bases considered was 272,391.

^a^FP = False Positives; FN = False Negatives; TP = True Positive; TN = True Negatives.

### Removal of non-homopolymer associated indel errors

From both the 6 training and 17 test samples, the majority of the false positive indels called prior to QD-VARW filtering were located in homopolymers (Additional file 1: Figure S5). However, some false positives were also detected in non-homopolymer regions. (Additional file 1: Figure S5). These non-homopolymer-associated errors have also been reported elsewhere [12]. In our 23 samples, application of the QD filter to putative indels detected by the TS3.4 + SAMtools workflow removed 75% of the non-homopolymer-associated errors, thus demonstrating the usefulness of the QD filter in minimising such errors.

## Discussion

The advent of NGS technology has increased sequencing capacity and lowered the cost of sequencing [21], making it an appealing alternative to Sanger sequencing for genetic testing. In particular, the commercial availability of benchtop sequencers since the launching of PGM by Life Technology in 2011 [7], has attracted interest from clinical laboratories [22].

A workflow for clinical *BRCA1* and *BRCA2* diagnosis using PGM sequencing was recently proposed and evaluated in [2]. The analysis workflow was designed to detect both single nucleotide substitutions and microindels. It was based on TS2.0 variant calls followed by several filters, including a filter to consider only variants found at frequencies < 15% in the tested population. The SNV calling using this workflow was impressive, with a100% sensitivity and an FDR of 1/4 or 25% (data from Tables three and four in [2]) when polymorphisms were not included in the evaluation. For microindels, this pipeline had on average one false discovery per sample, with an FDR of 20/23 or 87% (data from Tables three and four in [2]). This is a high rate for clinical diagnosis [23–25].

The overall performance of SNV detection using the proprietary workflows was less problematic than indel detection in our study (Additional file 1: Tables S2 and S3). Generally, the TS workflows perform better in terms of sensitivity in which 100% sensitivity were achieved. But TS workflows generated more false positive SNVs, with an FDR as high as 21.43% as compared to workflows using SAMtools and GATK as variant callers, an estimate comparable to that of the previous finding [2].

Therefore we focused entirely on evaluating the sensitivity and specificity of indel detection due to the high FDR in the previous study [2]. Our study further investigated the characteristics of the indel errors and then developed a simple workflow that combines either the TMAP-TS3.4 or BWA with the SAMtools variant caller. These workflows achieved higher sensitivity and specificity than the TS workflows or the workflow reported by Costa *et al.* in reference [2]. For our combined training and test data, the FDR for TMAP-TS3.4 and SAMtools was 2/7 (29%) and 4/9 (44%) for BWA and SAMTools.

Despite the improved FDRs of indel detection, the rates remain relatively high using the TMAP-TS3.4 and SAMtools as well as the BWA and SAMtools workflows for clinical genetic testing. With a large sample size, more systematic false positives will likely be found in multiple samples. Unfortunately, it is challenging to eliminate these false positives by defining a threshold based on mutation frequency of these samples. Interestingly, we observed a false positive indel filtered by QD_th_ in one sample that marginally escaped filtering in another sample. We thus proposed to remove indels detected in a specific sample that were also found in the set of indels filtered by QD_th_ and VARW_th_ from other samples. Using this strategy, we managed to eliminate an additional false positive from each workflow without compromising sensitivity, achieving an FDR of 1/6 (17%) for TMAP-TS3.4 and SAMtools, and 3/8 (38%) for BWA and SAMTools. Nonetheless, larger sample sizes and additional sample sets that have common true positive indels will be required in order to test the performance of this strategy.

## Conclusions

**Figure 3:**
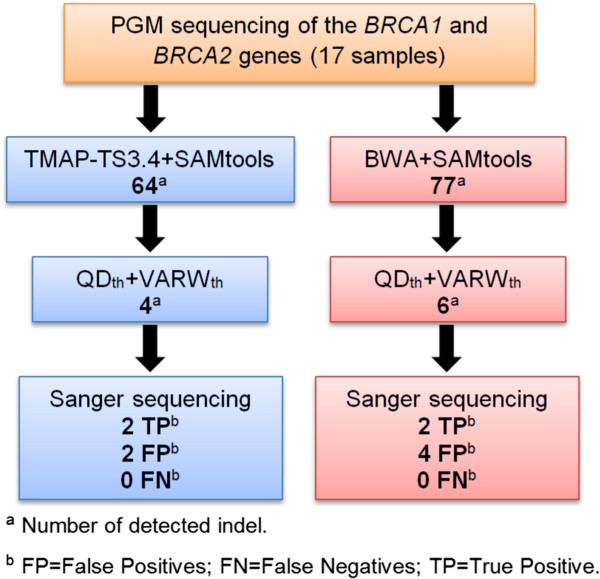
**Proposed workflows for highly sensitive and specific indel detection from PGM data.** BAM files were generated by read alignment of PGM sequencing outputs using either TMAP-TS3.4 (blue) or BWA (red). SAMtools was used to call indels. This was followed by a post-indel calling filtering using QD_th_ and VARW_th_. An independent confirmation of called indel was performed using Sanger sequencing. The numbers of indels called by each step were specified.

The newer versions of TS have shown improvements in both the alignment and variant calling performance, which in turn increased indel calling sensitivity and specificity. However, even the very recent TS variant caller (TS3.4) had a lower sensitivity than the GATK or SAMtools variant callers. Here, we present a computational workflow that (1) uses the TS3.4 or BWA as the read mapper (2) SAMtools as the variant caller and (3) VARW_th_ and QD_th_ as post-variant-calling filters. This workflow resulted in indel detection with overall 100% sensitivity, ≥ 99.99% specificity and ≤ 44% FDR of all 23 samples (Tables 2 and 3; Figure 3). Our findings demonstrate that a significant reduction of the false positives can be achieved with an effective computational indel calling workflow. Nevertheless, the wide range of confidence intervals due to the small sample size in this study suggests that a larger data with known true indels will be required for achieving a more conclusive estimation of the sensitivity and FDR.

## Availability of supporting data

Raw sequence data has been submitted to the European Nucleotide Archive (http://www.ebi.ac.uk/ena/) under accession number PRJEB5466.

## Electronic supplementary material

Additional file 1: Table S1: Adjusted indel calling parameters of TSVC3.4 to achieve maximum detection sensitivity. **Table S2.** Comparison of SNV calling in the 6 training samples using different variant calling workflows. **Table S3.** Comparison of SNV calling in the 17 additional validation-set using different workflows. **Figure S1.** IGV snapshot of read alignments at a region that includes the position of a false negative indel specific to TS2.0 and TS2.2 indel calling (*BRCA2:NM_000059:c.3846_3847del*). **Figure S2.** IGV snapshot of read alignments at a region that includes the position of a false negative indel generated by TS2.0, TS2.2 and TS3.4 indel calling (*BRCA2:NM_000059:c.7696_7697insA*). **Figure S3.** MAPQ distributions at the position of a false negative indel specific to TS2.0 and TS2.2 indel calling (*BRCA2:NM_000059:c.3846_3847del*). **Figure S4.** MAPQ distributions at the position of false negative indel generated by TS2.0, TS2.2 and TS3.4 indel calling (*BRCA2:NM_000059:c.7696_7697insA*). **Figure S5.** Distribution of homopolymer run length (HRun) associated with true (T) and false (F) positive indels. (DOCX 459 KB)
