# Supplementary material for: Evaluation and optimisation of indel detection workflows for ion torrent sequencing of the BRCA1 and BRCA2 genes
Source: BMC Genomics. 2014 Jun 24;15(1):516. doi: 10.1186/1471-2164-15-516 (PMC4079958; doi:10.1186/1471-2164-15-516)
Supplement: Supplementary file 1 — Additional file 1: Table S1: Adjusted indel calling parameters of TSVC3.4 to achieve maximum detection sensitivity. Table S2. Comparison of SNV calling in the 6 training samples using different variant calling workflows. Table S3. Comparison of SNV calling in the 17 additional validation-set using different workflows. Figure S1. IGV snapshot of read alignments at a region that includes the position of a false negative indel specific to TS2.0 and TS2.2 indel calling (BRCA2:NM_000059:c.3846_3847del). Figure S2. IGV snapshot of read alignments at a region that includes the position of a false negative indel generated by TS2.0, TS2.2 and TS3.4 indel calling (BRCA2:NM_000059:c.7696_7697insA). Figure S3. MAPQ distributions at the position of a false negative indel specific to TS2.0 and TS2.2 indel calling (BRCA2:NM_000059:c.3846_3847del). Figure S4. MAPQ distributions at the position of false negative indel generated by TS2.0, TS2.2 and TS3.4 indel calling (BRCA2:NM_000059:c.7696_7697insA). Figure S5. Distribution of homopolymer run length (HRun) associated with true (T) and false (F) positive indels. (DOCX 459 KB) [file 12864_2013_6187_MOESM1_ESM.docx]

**Additional file 1**

**Table S1. Adjusted indel calling parameters of TSVC3.4 to achieve maximum detection sensitivity.**

| **Default TSVC3.4** | **Adjusted TSVC3.4** |
| --- | --- |
| **Indel Caller Parameter Settings:** | **Indel Caller Parameter Settings:** |
| **min_allele_frequency=0.2** | **min_allele_frequency=0** |
| **min_indel_count_for_genotyping=5** | **min_indel_count_for_genotyping=0** |
| hp_max_single_peak_std_23=18 | hp_max_single_peak_std_23=18 |
| hp_max_length=9 | hp_max_length=9 |
| info_vcf=0 | info_vcf=0 |
| hp_min_cov_each_strand=3 | hp_min_cov_each_strand=3 |
| **min_mapping_quality_score=4** | **min_mapping_quality_score=0** |
| hp_stb_max_two_peaks_relative_bias=0.8 | hp_stb_max_two_peaks_relative_bias=0.8 |
| hp_max_single_peak_std_increment=5 | hp_max_single_peak_std_increment=5 |
| hp_stb_fpe_min_coverage=30 | hp_stb_fpe_min_coverage=30 |
| hp_stb_max_avg_peak_srand_distance=40 | hp_stb_max_avg_peak_srand_distance=40 |
| downsample_to_coverage=2000 | downsample_to_coverage=2000 |
| hp_low_stringency=0 | hp_low_stringency=0 |
| fpe_max_peak_deviation=31 | fpe_max_peak_deviation=31 |
| **Long Indel Assembly Parameter Settings:** | **Long Indel Assembly Parameter Settings:** |
| kmer_len=19 | kmer_len=19 |
| min_var_count=5 | min_var_count=5 |
| max_hp_length=8 | max_hp_length=8 |
| relative_strand_bias=0.80 | relative_strand_bias=0.80 |
| min_var_freq=0.15 | min_var_freq=0.15 |
| min_indel_size=4 | min_indel_size=4 |
| short_suffix_match=5 | short_suffix_match=5 |
| **Filter-Indels Parameter Settings:** | **Filter-Indels Parameter Settings:** |
| bay-score-minlen=11 | bay-score-minlen=11 |
| **min-bayesian-score=2.5** | **min-bayesian-score=0** |
| **variant-strand-bias=0.90** | **variant-strand-bias=1** |
| **min-var-freq=0.199** | **min-var-freq=0** |

Parameters that were adjusted for maximum sensitivity are shown in red and bold.

**Table S2. Comparison of SNV calling in the 6 training samples using different variant calling workflows.**

| **Read mapper** | **Variant caller** | **FP**^a^ | **FN**^a^ | **TP**^a^ | **TN**^a^ | **Sensitivity [95% CI]** | **Specificity [95% CI]** | **FDR [95% CI]** |
| --- | --- | --- | --- | --- | --- | --- | --- | --- |
| TMAP-TS2.0 | TSVC2.0 | 4 | 1 | 32 | 96101 | 96.97% [86.7, 99.67] | 99.99% [99.99, 100] | 11.11% [3.87, 24.29] |
| TMAP-TS2.2 | TSVC2.2 | 4 | 0 | 33 | 96101 | 100% [94.39, 100] | 99.99% [99.99, 100] | 10.81% [3.76, 23.69] |
| TMAP-TS3.4 | TSVC3.4 | 9 | 0 | 33 | 96096 | 100% [94.39, 100] | 99.99% [99.98, 100] | 21.43% [11.17, 35.47] |
| TMAP-TS2.0 | GATK | 0 | 1 | 32 | 96105 | 96.97% [86.7, 99.67] | 100% [100, 100] | 0% [0, 5.78] |
| TMAP-TS2.2 | GATK | 0 | 2 | 31 | 96105 | 93.94% [81.95, 98.72] | 100% [100, 100] | 0% [0, 5.96] |
| ^*^TMAP-TS3.4 | GATK | 0 | 3 | 30 | 96105 | 90.91% [77.69, 97.37] | 100% [100, 100] | 0% [0, 6.15] |
| TMAP-TS2.0 | SAMtools | 0 | 2 | 31 | 96105 | 93.94% [81.95, 98.72] | 100% [100, 100] | 0% [0, 5.96] |
| TMAP-TS2.2 | SAMtools | 0 | 1 | 32 | 96105 | 96.97% [86.7, 99.67] | 100% [100, 100] | 0% [0, 5.78] |
| ^*^TMAP-TS3.4 | SAMtools | 1 | 0 | 33 | 96104 | 100% [94.39, 100] | 99.99% [99.99, 100] | 2.94% [0.32, 12.93] |
| ^*^BWA | GATK | 0 | 1 | 32 | 96105 | 96.97% [86.7, 99.67] | 100% [100, 100] | 0% [0, 5.78] |
| ^*^BWA | SAMtools | 0 | 1 | 32 | 96105 | 96.97% [86.7, 99.67] | 100% [100, 100] | 0% [0, 5.78] |

We considered all bases in coding exons. Across the 6 samples the total number of bases considered was 96,138.

^a^ FP=False Positives; FN=False Negatives; TP=True Positive; TN=True Negatives.

**Table S3. Comparison of SNV calling in the 17 additional validation-set using different workflows.**

| **Read mapper** | **Variant caller** | **FP**^a^ | **FN**^a^ | **TP**^a^ | **TN**^a^ | **Sensitivity [95% CI]** | **Specificity [95% CI]** | **FDR [95% CI]** |
| --- | --- | --- | --- | --- | --- | --- | --- | --- |
| TMAP-TS3.4 | GATK | 4 | 9 | 116 | 272262 | 92.8% [87.27, 96.38] | 99.99% [99.99, 100] | 3.33% [1.14, 7.73] |
| TMAP-TS3.4 | SAMtools | 4 | 7 | 118 | 272262 | 94.4% [89.32, 97.46] | 99.99% [99.99, 100] | 3.28% [1.12, 7.61] |
| BWA | GATK | 5 | 14 | 111 | 272261 | 88.8% [82.4, 93.43] | 99.99% [99.99, 100] | 4.31% [1.66, 9.19] |
| BWA | SAMtools | 3 | 9 | 116 | 272263 | 92.8% [87.27, 96.38] | 99.99% [99.99, 100] | 2.52% [0.72, 6.57] |

We considered all bases in coding exons. Across the 17 samples the total number of bases considered was 272,391.

^a^ FP=False Positives; FN=False Negatives; TP=True Positive; TN=True Negatives.


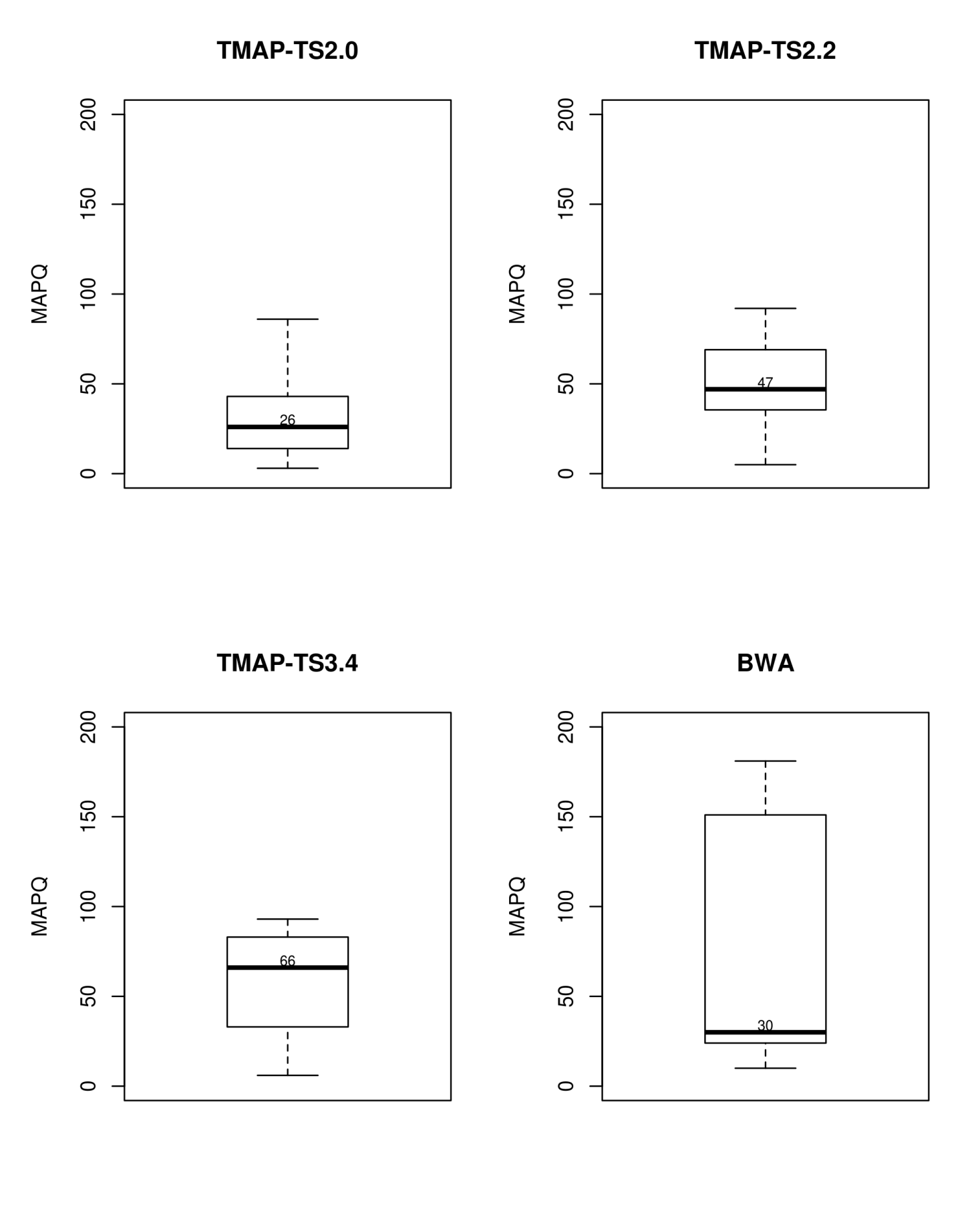


**Figure S1**. MAPQ distributions at the position of a false negative indel specific to TS2.0 and TS2.2 indel calling (*BRCA2:NM_000059:c.3846_3847del*). Each panel represents the MAPQ distribution of aligned reads (generated by corresponding read mapper, TMAP) that include the indel. More reads with lower MAPQ scores were found in the alignments generated by TS2.0 and TS2.2. A higher diversity of MAPQ scores was generated by BWA.


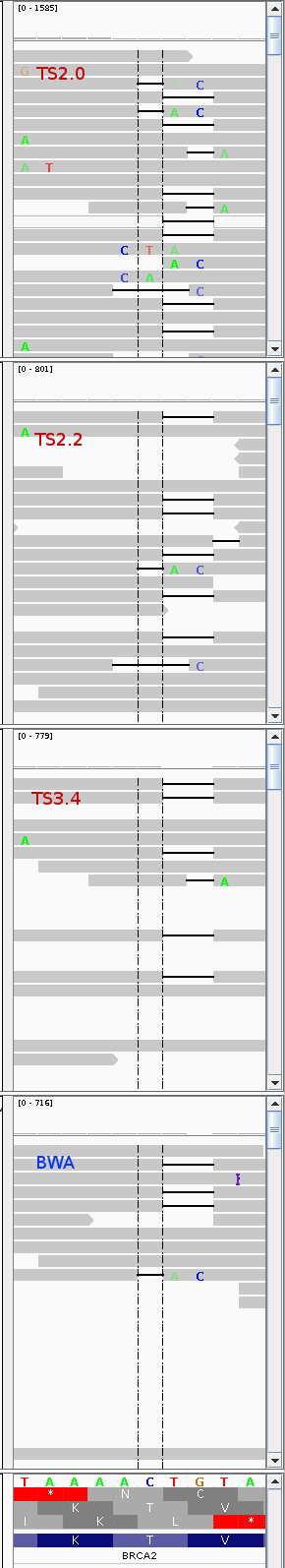


**Figure S2.** IGV snapshot of read alignments at a region that includes the position of a false negative indel specific to TS2.0 and TS2.2 (*BRCA2:NM_000059:c.3846_3847del*). The four panels show read alignments generated by TS2.0, TS2.2, TS3.4 and BWA (top to bottom). The lengths for the gaps of the top two panels (TS2.0 and TS2.2) were more diverse than the bottom two panels (TS3.4 and BWA); multiple gaps were also observed in adjacent positions of the indel for the top two panels, indicating that the quality of read alignment might affect the detection of this specific indel.


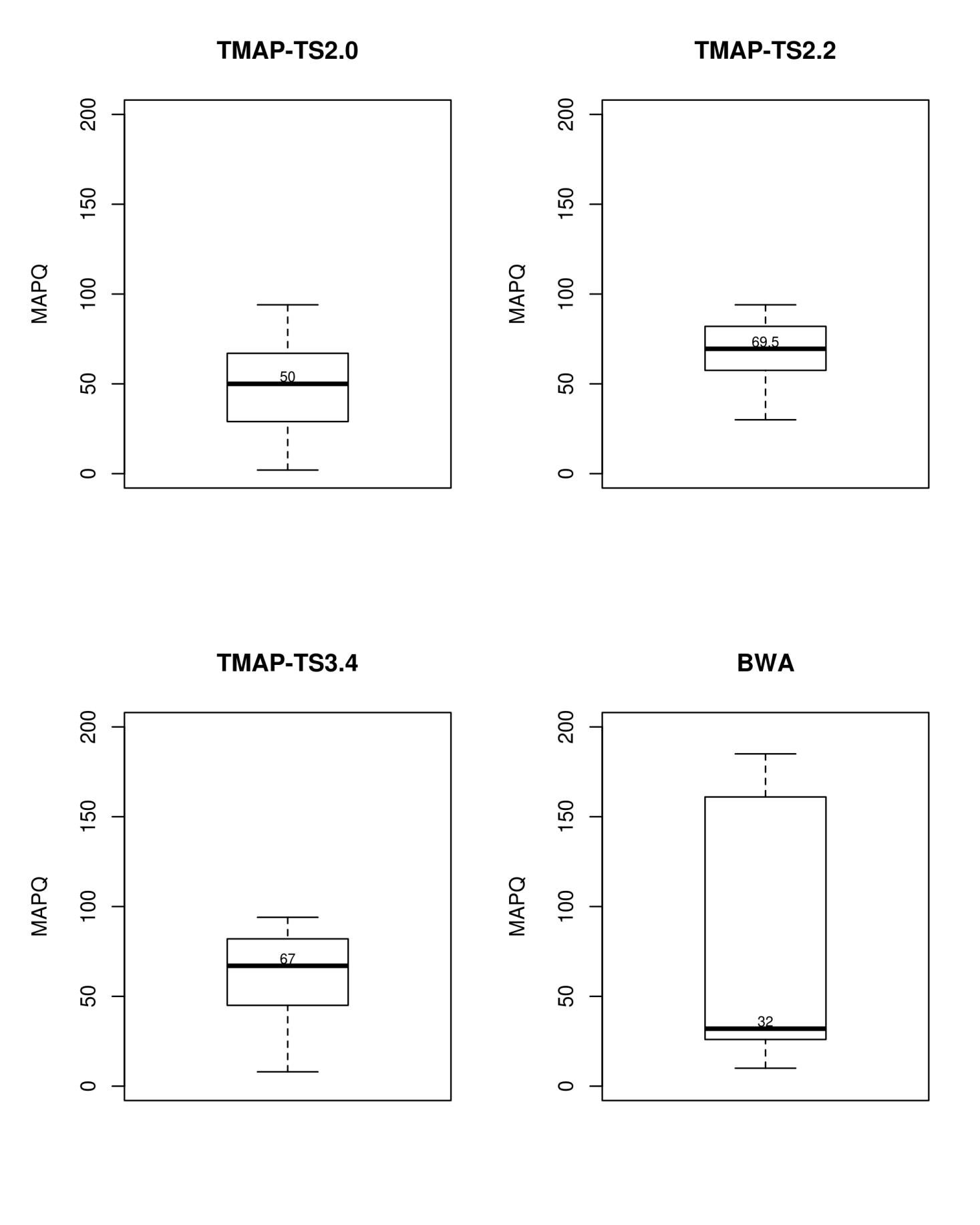


**Figure S3.** MAPQ distributions at the position of false negative indel generated by TS2.0, TS2.2 and TS3.4 indel calling (*BRCA2:NM_000059:c.7696_7697insA*). Each panel represents the MAPQ distribution of aligned reads (generated by corresponding read mapper, TMAP) that include the indel. The medians for the MAPQ distributions generated by TS2.2 and TS3.4 were similar. The distributions generated by TS2.2 and TS3.4 were comparable to the distribution generated by TS3.4 in Figure S3, suggesting that their alignment quality should be sufficient for indel calling. A higher diversity of MAPQ scores was generated by BWA.


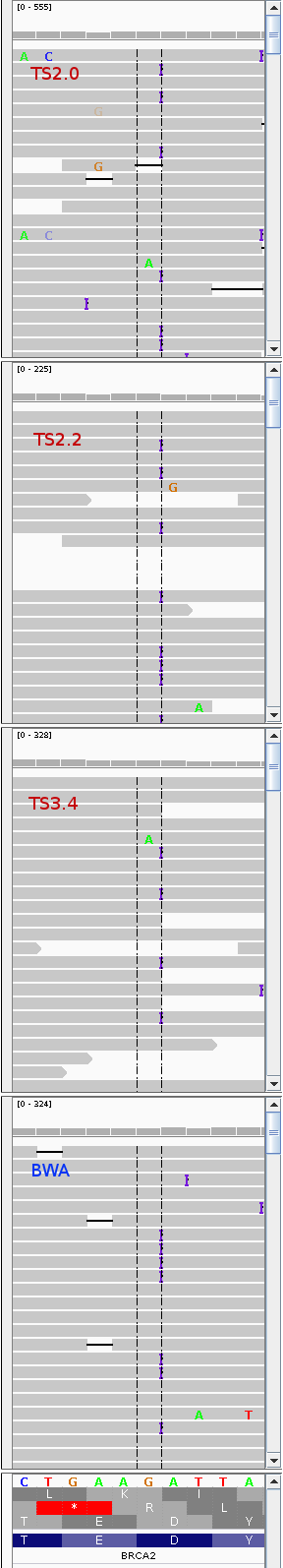


**Figure S4**. IGV snapshot of read alignments at a region that includes the position of a false negative indel generated by TS2.0, TS2.2 and TS3.4 (*BRCA2:NM_000059:c.7696_7697insA*). The four panels show read alignments generated by TS2.0, TS2.2, TS3.4 and BWA (top to bottom). All panels have similar insert length distribution, non-reference allele frequency and alignment profile at proximal regions, indicating that the false negative indel is unlikely to be due to low quality of read alignment.

**Figure S1**. MAPQ distributions at the position of a false negative indel specific to TS2.0 and TS2.2 indel calling (*BRCA2:NM_000059:c.3846_3847del*). Each panel represents the MAPQ distribution of aligned reads (generated by corresponding read mapper, TMAP) that include the indel. More reads with lower MAPQ scores were found in the alignments generated by TS2.0 and TS2.2. A higher diversity of MAPQ scores was generated by BWA.

**Figure S3.** MAPQ distributions at the position of false negative indel generated by TS2.0, TS2.2 and TS3.4 indel calling (*BRCA2:NM_000059:c.7696_7697insA*). Each panel represents the MAPQ distribution of aligned reads (generated by corresponding read mapper, TMAP) that include the indel. The medians for the MAPQ distributions generated by TS2.2 and TS3.4 were similar. The distributions generated by TS2.2 and TS3.4 were comparable to the distribution generated by TS3.4 in Figure S3, suggesting that their alignment quality should be sufficient for indel calling. A higher diversity of MAPQ scores was generated by BWA.


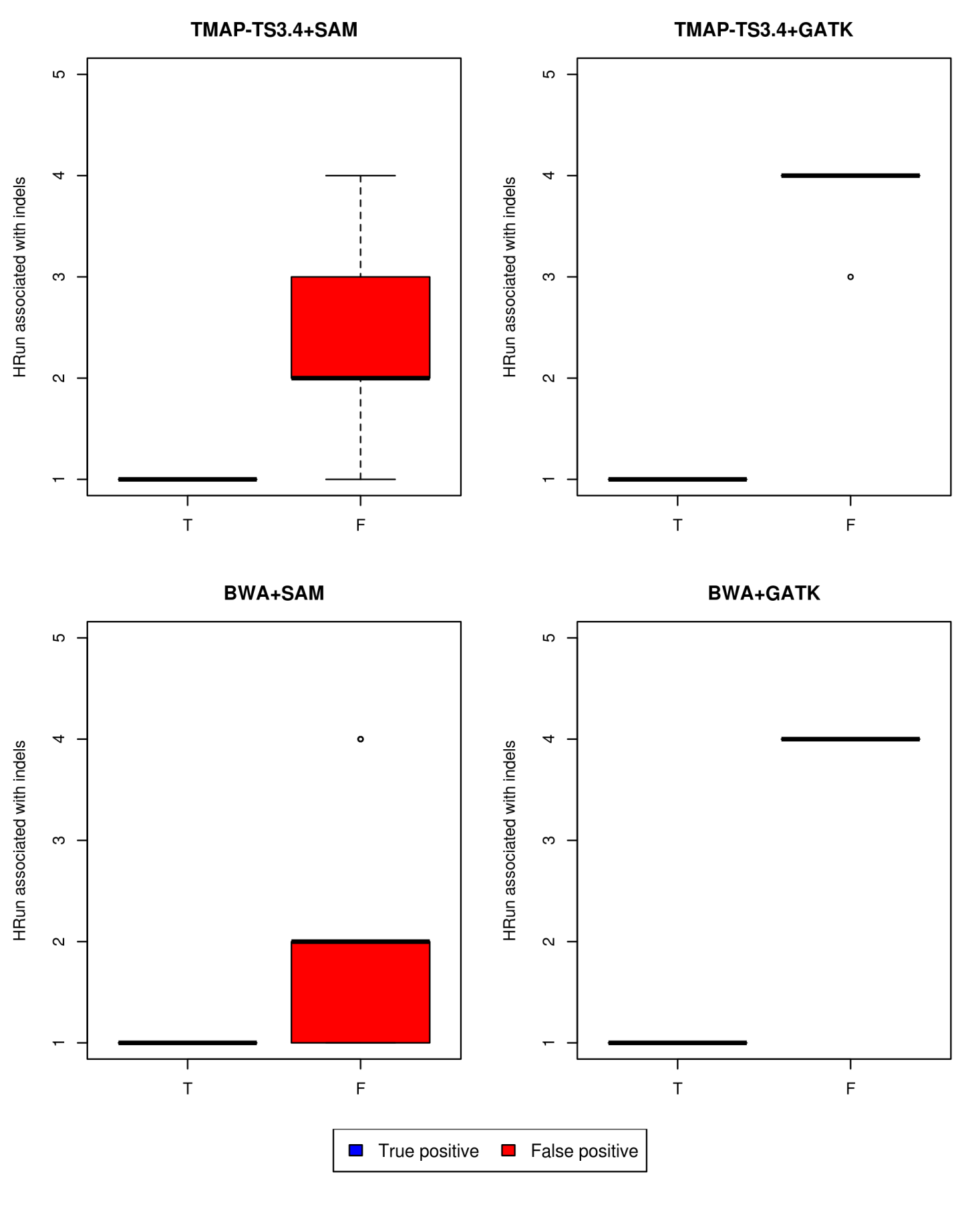


**Figure S5.** Distribution of homopolymer run length (HRun) associated with true (T) and false (F) positive indels. Each of the five panels shows two distributions of homopolymer run length for true (blue) and false (red) positive indels. Each panel corresponds to one indel calling workflow. All panels suggest that false positive indels are associated with the longer homopolymer run length. Two panels that show indel calling with SAMtools (TMAP-TS3.4+ SAM, BWA+ SAM) include false positive indels located at the singleton (HRun=1) region.
